# Treatment of neovascular age-related macular degeneration: one year real-life results with intravitreal Brolucizumab

**DOI:** 10.3389/fmed.2024.1467160

**Published:** 2025-01-15

**Authors:** Settimio Rossi, Carlo Gesualdo, Ernesto Marano, Raffaele Perrotta, Maria Consiglia Trotta, Antonio Del Giudice, Francesca Simonelli

**Affiliations:** ^1^Eye Clinic, Multidisciplinary Department of Medical, Surgical and Dental Sciences University of Campania “Luigi Vanvitelli”, Naples, Italy; ^2^Eye Unit, G. Rummo Hospital, Benevento, Italy; ^3^Department of Experimental Medicine, University of Campania "Luigi Vanvitelli", Naples, Italy

**Keywords:** age-related macular degeneration, intravitreal injection, brolucizumab, real-life, neovascularization

## Abstract

**Background:**

Age-related macular degeneration (AMD) is a prevalent cause of irreversible vision loss worldwide, particularly among the elderly population. Two forms of late AMD are described: neovascular AMD (nAMD), characterized by abnormal choroidal blood vessel growth, and atrophic (dry) AMD, involving retinal cell degeneration. Intravitreal anti-vascular endothelial growth factor (anti-VEGF) agents have transformed nAMD treatment, with Brolucizumab emerging as a promising therapy. The aim of this study is to provide the real-life anatomical-functional and safety results, after 1 year of treatment experience with Brolucizumab.

**Methods:**

This is a retrospective observational real-life study in which 44 patients (44 eyes) diagnosed with nAMD and treated with Brolucizumab were enrolled. We identified two groups: group 1 (24 treatment-naïve eyes) that received a loading dose of 3 monthly intravitreal injections of Broluciziumab 6 mg (0.05 mL solution) + Q8w/Q12w regimen, and a Group 2 (20 non-naïve eyes) which performed 1 injection + ProReNata (PRN) scheme. Monthly, all participants underwent comprehensive ophthalmological evaluation until 12 months follow-up.

**Results:**

We observed a significant improvement in best corrected visual acuity (39 ± 15 L vs. 30 ± 17 L; *p* < 0.01) and central retinal thickness (265 ± 89 *μ* vs. 360 ± 129 μ; *p* < 0.0001) at the end of follow-up without any differences between treatment-naïve and non-naïve patients. These results were obtained with a low number of injections (3.7 ± 1.9) with only one case of intraocular drug-related adverse event. Finally, the presence of subretinal hyperreflective material correlates with lower visual recovery.

**Discussion:**

Our findings highlight the efficacy of Brolucizumab in managing wet-AMD and suggest its role for long-term efficacy in stabilizing retinal exudation and fluid accumulation, resulting in improved visual prognosis.

## 1 Introduction

Age-related macular degeneration (AMD) is a multifactorial, progressive, and debilitating disease predominantly affecting the elderly population, representing a significant public health concern worldwide (1). It stands as one of the leading causes of irreversible blindness in industrialized nations, imposing substantial socioeconomic burdens on healthcare systems and individuals alike (2, 3). AMD causes a progressive threat to visual function, particularly in its advanced stages, where it manifests as a degenerative condition primarily affecting the macula, the central region of the retina responsible for distinct vision (1). AMD is clinically stratified into three main stages: early, intermediate, and late (4). The early and intermediate stages are often asymptomatic or associated with mild visual symptoms, characterized by the presence of typical hallmarks such as drusen deposits and pigmentary changes within the macula (5). These stages are marked by progressive neuroretinal degeneration. Late-stage AMD, on the other hand, presents a more advanced and debilitating clinical picture, with two primary phenotypic manifestations: neovascular (wet) AMD and atrophic (dry) AMD. Neovascular AMD is characterized by the aberrant growth of choroidal blood vessels into retinal layers or below them, leading to the formation of abnormal vascular networks in the macular region (6). That process compromises retinal integrity and function, resulting in profound visual impairment. Conversely, atrophic AMD is distinguished by progressive degeneration of retinal pigment epithelial cells and photoreceptors, resulting in geographic atrophy within the macular area (7). Both forms of late AMD contribute significantly to vision loss and pose considerable challenges for effective management and treatment.

Historically, the therapeutic landscape for wet AMD has evolved considerably, with treatment paradigms transitioning from conventional modalities such as thermal laser photocoagulation and photodynamic therapy to the advent of intravitreally anti-vascular endothelial growth factor (anti-VEGF) agents (8). The introduction of intravitreal anti-VEGF therapy heralded a paradigm shift in AMD management, offering unprecedented efficacy in preserving visual function and improving quality of life, delaying disease progression (9, 10). Notably, numerous landmark clinical trials have demonstrated the efficacy and safety of anti-VEGF agents, consolidating their position as first-line treatments for neovascular AMD (11). Currently approved anti-VEGF agents include ranibizumab, aflibercept, brolucizumab, and faricimab (bispecific antibody). Bevacizumab is also an anti-VEGF agent used off-label to manage neovascular AMD (nAMD). However, there are no direct comparison studies that show the superiority of one drug over another, but only noninferiority trials that compare different anti-VEGF agents. For example, in a large noninferiority trial by the CATT (Center for Preventive Ophthalmology and Biostatistics) research group with a large number of patients enrolled (1208), Bevacizumab and Ranibizumab, both administered monthly, showed the same results after 1 year follow-up (8.0 and 8.5 letters gained, respectively). Moreover, authors highlighted also how Bevacizumab as needed was equivalent to Ranibizumab as needed with +5.9 and + 6.8 letters gained, respectively (12). Authors also analyzed the results of re-randomizing the same patients into continuous monthly treatment and as-needed treatment at year 2, discovering that the as-needed group lost −1.8 letters compared with those undergoing monthly treatment (13). Those two studies highlighted how monthly treatment approach, even being more expensive for healthcare systems and stressful for patients, is more effective in maintaining visual acuity in time.

However, despite the remarkable therapeutic advancements achieved with anti-VEGF therapy, several challenges persist in the management of AMD, particularly in the context of real-world clinical practice (14). The burden of frequent intravitreal injections, the need for long-term monitoring, and possible treatment-related complications pose significant challenges for both patients and healthcare systems to optimize AMD treatment strategies. In recent years, efforts have focused on optimizing treatment protocols and exploring novel therapeutic modalities aimed at enhancing treatment efficacy and reducing treatment burden (15). Brolucizumab, a novel anti-VEGF agent, has gained significant attention as a potential candidate for advancing the treatment paradigm in neovascular AMD (16–18). As a single-chain antibody fragment targeting all isoforms of VEGF-A, Brolucizumab offers the potential for enhanced potency and sustained therapeutic effect (19). Its small molecular size facilitates higher drug concentrations within the vitreous cavity following intravitreal administration, thereby optimizing retinal bioavailability, and minimizing treatment frequency (20). Dealing with its small molecular size, some authors have hypothesized that, even if brolucizumab should be less immunogenic because of an Fc portion missing in its biomolecular anatomy, actually it can be more immunogenic because of its size and potentiality to unfold and consequently expose epitopes that may not be identified by the immune system (21).

Clinical trials evaluating the efficacy and safety of Brolucizumab have yielded promising results, demonstrating non-inferiority to existing anti-VEGF agents in terms of visual acuity outcomes and anatomical improvements (22). Notably, Brolucizumab has shown favorable outcomes in reducing central retinal thickness and extending treatment intervals, offering the prospect of reduced treatment burden and improved patient convenience (23). In fact, in HAWK and HARRIER trials, treatment regimen was made up by a loading phase of 3 monthly injections followed by a q8w/q12w re-treatment scheme based on disease activity. Notably, the authors reported that a high percentage of patients, 55.6% in HAWK and 51.0% in HARRIER, maintained q12w dosing after loading until the end of the follow-up (48 week). Furthermore, authors observed that in patients without disease activity in the first 12 weeks, the probability of maintaining q12w scheme thought-out 48 weeks was very high: 85.4% in HAWK and 81.7% in HARRIER (16). Interestingly, authors have also reported that in patients with nAMD characterized by refractory residual retinal fluid despite undergoing repeated intravitreal injections of anti-VEGF, switching from other drugs to Brolucizumab can help in obtaining both functional and anatomical improvement (24).

Another relevant consideration regarding visual recovery and treatment scheme after loading phase is the evaluation of a new optical coherence tomography (OCT) biomarker highlighted by Sadda et al. in a recent post-hoc analysis (25), that is the subretinal hyperreflective material (SHRM), corresponding to the accumulation of blood, fibrin and fibro-scar tissue in the subretinal space. SHRM creates a mechanical barrier between the retina and retinal pigment epithelium (RPE), thus interfering with metabolic exchanges and normal functionality of the photoreceptors, causing worse functional improvement during anti-VEGF intravitreal therapy. On this regard, Sadda et al. observed that high SHRM thickness postloading and high SHRM thickness variability over time are negative predictive factors in patients with nAMD. Moreover, the authors described an higher percentage SHRM thickness reductions from baseline in patients treated with Brolucizumab versus Aflibercept (25).

However, concerns regarding the incidence of treatment-related adverse events, including intraocular inflammation and retinal vasculitis, have been raised, necessitating careful consideration of safety profiles in clinical practice (26). In a *post hoc* review of the HAWK and HARRIER study authors analyzed the incidence of adverse events correlated with intravitreal administration of Brolucizumab, carefully excluding forms of intraocular inflammation (IOI) or endophthalmitis not related to the drug. They found out that the incidence of definite/probable IOI was 4.6, IOI + retinal vasculitis was 3.3%, and IOI + retinal vasculitis + retinal occlusion was 2.1%. In brolucizumab-treated eyes with definite/probable IOI, the risk of at least moderate visual acuity loss was 22.2% in eyes with signs of retinal vasculitis and 30.4% in eyes with signs of retinal vasculitis + retinal occlusion; the risk of severe visual acuity loss was 13.9% in eyes with signs of retinal vasculitis and 21.7% in eyes with signs of retinal vasculitis + retinal occlusion (27). In a large retrospective analysis (432,794 injections) is reported that severe IOI occurred at a rate of 1.06/1000 aflibercept injections and 0.64/1000 ranibizumab injections (28). However, despite these data, authors reported that overall rates of moderate or severe visual acuity loss (including that associated with definite/probable IOI, retinal vasculitis and/or retinal occlusion) were similar for brolucizumab and aflibercept (7.4 and 7.7%, respectively). Based on these findings authors suggest accurate slit-lamp examination and ophthalmoscopy when treating patients with Brolucizumab, looking for subtle vasculitis and/or occlusive disease and/or signs of inflammation in the OCT scans (27). Furthermore, in a recent Japanese study, authors reported an association of macular atrophy and SHRM with the development of intravitreal injection-related IOI (21). Another aspect about Brolucizumab analyzed in recent literature is its relevant effect in reducing ocular blood flow in the 30 min following the injection. This reaction is provoked by other anti-VEGF as well and it is already clearly described in literature even with OCT evidence on the choroidal thickness (29) and it is thought to be caused by the activation of endothelial nitric oxide synthase (30). However, in a recent work by Kato et al., in 3 out of 10 patients studied, Brolucizumab decreased ocular blood flow at choroid of more than 30% and this reaction was not present in patients receiving intravitreal aflibercept, even if this finding was not correlated with any clinical significant variation (31).

Real-world evidence, especially Italian, relating to the long-term efficacy and safety of Brolucizumab remains limited, highlighting the need for further research and post-marketing surveillance to elucidate its clinical utility and risk–benefit profile (32, 33). Therefore, the aim of this study is to provide the real-life anatomical-functional and safety results, after 1 year of treatment experience with Brolucizumab.

## 2 Materials and methods

This is a retrospective observational study in which 44 patients (44 eyes) diagnosed with neovascular age-related macular degeneration (nAMD) were enrolled and treated. Patients were followed both at the Ophthalmology Clinic of the University of Campania “Luigi Vanvitelli,” Naples, Italy, and at the Ophthalmology Unit of the “Rummo” Hospital in Benevento, Italy. Table 1 summarizes the demographic and clinical data of the included subjects. The study adhered to the principles outlined in the Declaration of Helsinki and received the approval of the Institutional Board of Auditors of the University of Campania “Luigi Vanvitelli” (Prot. 0003239/I, 01/02/2023). Informed consent to participate in the study was obtained from all participants. We adopted the following exclusion criteria: previous treatments with Brolucizumab; concomitant ocular pathologies like diabetic retinopathy, hereditary retinal dystrophies, retinal vascular occlusions, uveitis; laser photocoagulation and/or vitrectomy performed in the study eye in the last 6 months.

**Table 1:** Demographic and clinical ophthalmic data of the enrolled patients.

Characteristics		Naïve	Non naïve
Age-mean (SD)	Years – 77 ± 5.3	76 (±5.2)	78 (±4.3)
Gender	Male: 25 (56.8%)	16 (64%)	9 (36%)
	Female: 19 (43.2%)	10 (43%)	9 (47%)
Systemic risk factors			
Hypertension		17	20
Smoking		11	14
Alcohol		3	4
Number of eyes		24 (55%)	20 (45%)
Lens status	Phakic: 16 (34%)		
	Pseudophakic: 28 (66%)		
MNV type	Type 1 (n. 28) (64%)	16 (57%)	12 (43%)
	Type 2 (n.12) (27%)	5 (41.7%)	7 (58.3%)
	Type 3 (n.4) (9%)	3 (75%)	1 (25%)

### 2.1 Study design

We divided the entire cohort into two groups: group 1 (24 treatment-naïve eyes), which underwent a loading dose of 3 monthly intravitreal injections of Broluciziumab 6 mg (0.05 mL solution) + Q8w/Q12w regimen, and a Group 2 (20 non-naïve eyes) which underwent a treatment regimen of 1 injection + ProReNata (PRN) scheme. Monthly, all participants underwent comprehensive ophthalmological evaluation, including: Best-Corrected Visual Acuity (BCVA) assessment using Early Treatment Diabetic Retinopathy Study (ETDRS) chart at 2 m; anterior segment biomicroscopy and binocular indirect ophthalmoscopy performed with Haag-Streit slit lamp; Spectral-Domain Optical Coherence Tomography (SD-OCT) and Angiography-OCT (OCTA). SD-OCT imaging was conducted using Cirrus 6,000 Zeiss Engineering, employing the protocol Macular Cube 512 × 128. Central retinal thickness (CRT) was measured at the foveal center as the distance between the retinal surface and retinal pigment epithelium (RPE). The OCTA was also performed with Cirrus 6,000 Zeiss Engineering.

### 2.2 Study endpoints

The primary endpoints were: change in BCVA and CRT at 6 and 12 months. We also evaluated the incidence of adverse events such as vitreitis and/or vasculitis related to therapy with Brolucizumab. Additionally, patients were stratified on the basis of intraretinal (IR), subretinal (SR) and sub-RPE (SRPE) fluid distribution at baseline, in order to evaluate the visual change during follow-up for each subgroup analyzed.

Moreover, we analyzed the anatomical and functional outcomes considering also the type of macular neovascular membrane (MNV), classificating it as already widely described in literature (34):

- MNV type 1: characterized by a sub-RPE fibrovascular complex grown from the choriocapillaris into the sub-RPE space.
- MNV type 2: characterized by the presence of hyperreflective material originating for choriocapillaris, penetrating through the RPE and spreading subretinally.
- MNV type 3: characterized by proliferating vessels that extend from deep retinal capillary plexus towards the outer retina.

Finally, we evaluated the influence of SHRM on BCVA and its anatomical evolution during the 12 months follow up, as already described in a recent work by Sadda et al. (25).

Baseline and follow-up images were independently graded by two investigators (E.M., R.P.) and verified by a senior colleague (C.G).

### 2.3 Statistical analysis

Data distribution and homogeneity were assessed by using, respectively, Shapiro–Wilk test and Levene’s test. Student’s T-test was performed to compare 2 groups (for example, in the evaluation of injection number between naïve and non- naïve). Variables based on repeated observations were analyzed by Friedman’s test followed by Dunn’s *post hoc* test, or by repeated measures analysis of variance (RM-ANOVA) followed by Bonferroni’s multiple comparison test. Particularly, Mauchly’s test was performed to assess sphericity for RM-ANOVA, and if violated, Greenhouse–Geisser or Huynh-Feldt correction was applied. Pearson correlation analysis was performed to assess the strength of association between 2 variables, confirmed by a linear regression analysis. Data were reported as mean ± SD and a *p* value <0.05 was considered statistically significant.

## 3 Results

A collective of 44 eyes from a total sample size of 44 patients (effect size = 0.25; *α* value = 0.05, 1-*β* value = 0.95), comprising 25 males (56.8%) and 19 females (43.2%), affected by wet-AMD, were retrospectively included in this investigation. The mean age was 77 +/− 5.3 years. Notably, no substantial distinctions were highlighted between the two subgroups (naïve vs. non naïve) in terms of age and gender distribution. Regarding the type of MNV, patients were classified as follows: 64% type 1 MNV (n. 28); 27% type 2 MNV (n. 12); 9% type 3 MNV (n. 4). A comprehensive overview of systemic, functional and morphological features of the entire cohort is presented in Table 1.

An average number of 3.7 + 1.9 injections (IVTs) was performed in the entire cohort. Analyzing the 2 subgroups (Levene’s test: F statistic = 1.4222; *p* value = 0.2396), in naïve patients an average number of 3.4 + 1.3 IVTs was performed, while in non-naïve patients the average number of IVTs was 4.1 + 1.3 IVTs, without significant differences between the two subgroups in terms of number of IVTs (*p = 0.30*). Among naïve patients, 24 subjects (96%) maintained a Q12 regimen, performing a maximum of 2 IVTs after the initial loading dose, until the end of follow-up. However, among non-naïve patients, most patients (65%; 13 patients) performed 2 injections after the initial IVT.

### 3.1 Best-corrected visual acuity and optical coherence tomography results

**Figure 1:**
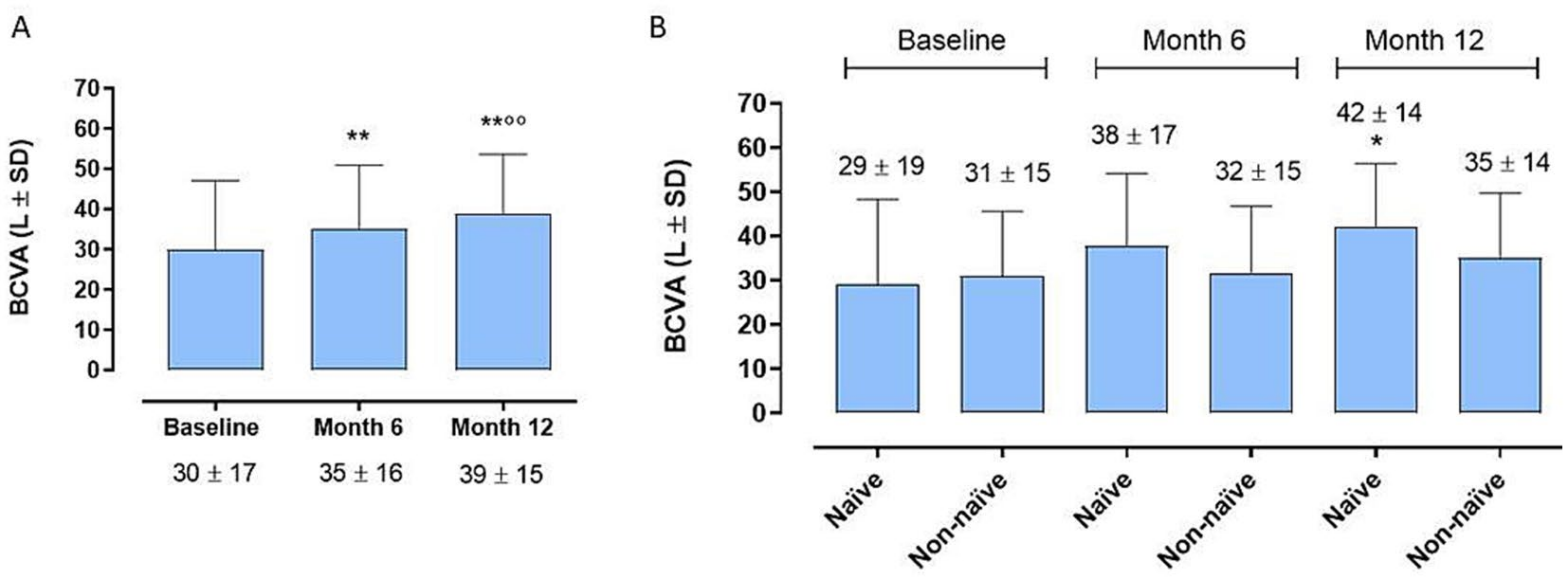
**(A)** Enhancements in best-corrected visual acuity (BCVA) at the 12-month follow-up relative to 6-month follow-up and to baseline [6-month mean difference (MD)]. ** *p* < 0.01 vs. Baseline; °° *p* < 0.01 vs. Month 6 (RM-ANOVA followed by Bonferroni’s test, Huynh-Feldt correction). **(B)** Best corrected visual acuity (BCVA) trends between naïve and non-naïve groups during the follow-up. * *p* < 0.01 vs. Baseline, same group; (RM-ANOVA, Greenhouse–Geisser correction).

**Figure 2:**
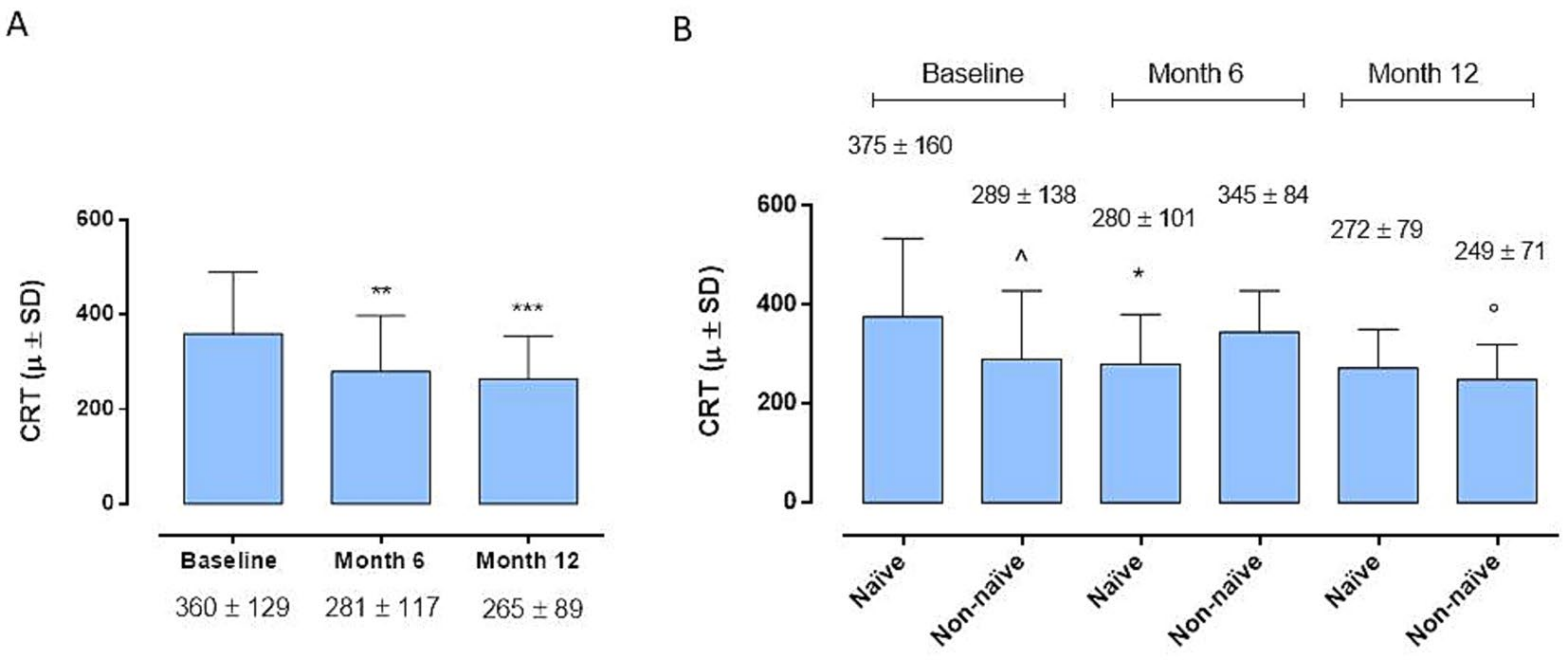
**(A)** Central retinal thickness (CRT) changes in the whole cohort. *p < 0.01 and ***p < 0.001 vs. Baseline (Friedman’s test, followed by Dunn’s test). **(B)** Central retinal thickness (CRT) changes between naïve and non-naïve groups during the follow-up (Friedman’s test, followed by Dunn’s test). *p < 0.05 vs Baseline, same group; °*p* < 0.05 vs Month 6, same group; ^*p* < 0.05 vs. naive at Baseline.

At the 6-month time point we observe a significant improvement in BCVA compared to baseline in the entire cohort (35 ± 16 letters (L) vs. 30 ± 17 L; *p < 0.01*) (Figure 1A), without significant differences between the 2 groups (38 ± 17 and 32 ± 15 L; *p > 0.05*) (Figure 1B). Instead, remarkably, the complete cohort exhibited statistically significant enhancements in BCVA at the 12-month follow-up relative to baseline (Figure 1A) (39 ± 15 L vs. 30 ± 17 L; *p < 0.01*). Interestingly, the disparity in BCVA improvement between Group 1 and Group 2 failed to obtain statistical significance at 12 months (42 ± 14 L vs. 35 ± 14 L; *p > 0.005*) (Figure 1B). Regarding CRT, marked improvements were observed in both groups, both at 6 and 12 months (281 ± 117 *μ* vs. 360 ± 129 μ, *p < 0.05*; 265 ± 89 μ vs. 360 ± 129 μ; *p < 0.001*) (Figure 2A), without significant variations between two groups at 6 and 12 months (*p > 0.05*) (Figure 2B).

### 3.2 Adverse events

Notably, our study reported only one case of acute-onset intermediate uveitis in a treatment-naïve male, whereas non-naïve patients remained free of adverse events throughout the follow-up period. The complete resolution of symptoms was reached with a treatment regimen involving local and systemic steroids and with mydriatic eye drop.

### 3.3 Best-corrected visual acuity changes and fluid distribution

**Figure 3:**
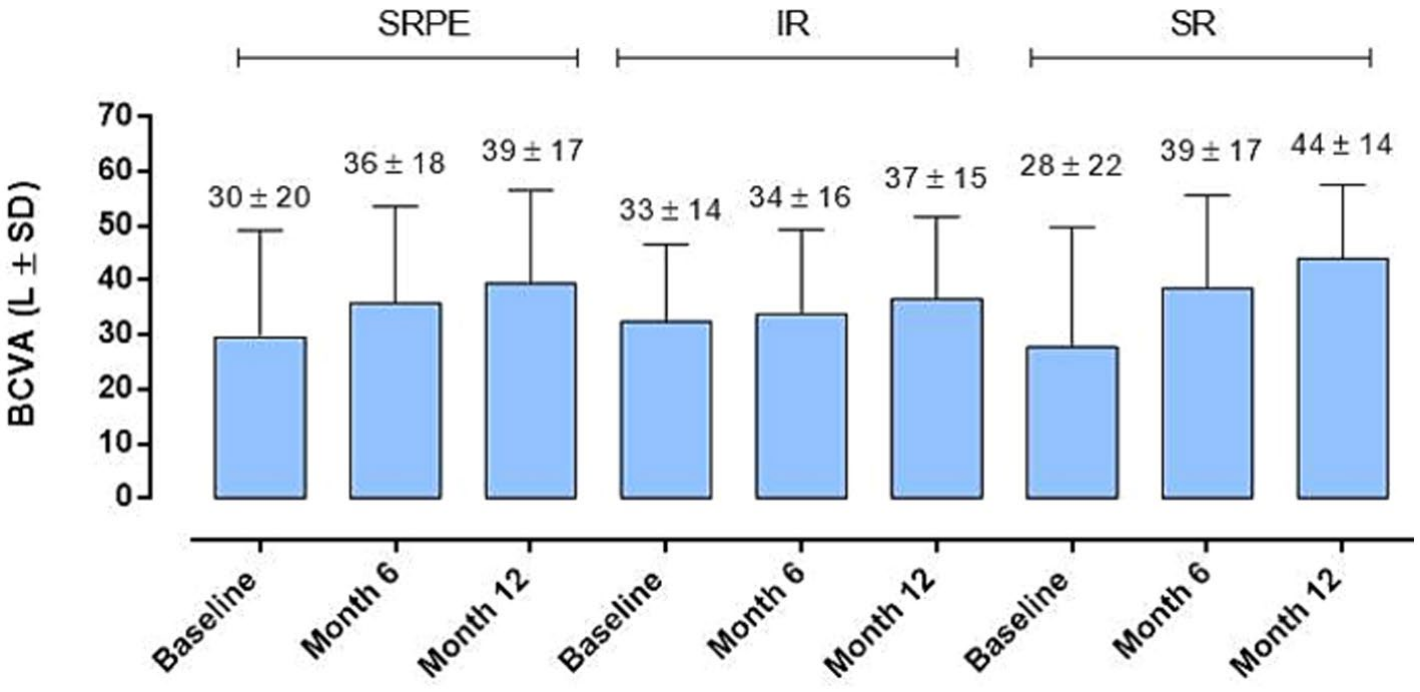
Best corrected visual acuity (BCVA) changes in relation to baseline fluid distribution. No significant difference was observed (RM-ANOVA, followed by Bonferroni’s test, Greenhouse–Geisser correction); intraretinal (IR), subretinal (SR) and sub- retinal pigment epithelium (SRPE).

By evaluating BCVA improvement in relation to the fluid distribution at baseline (IR, SR and SRPE), we did not observe a significant correlation between the localization of the fluids and the visual recovery at the various time points, without significant differences between the two groups of patients analyzed (*p > 0.05*) (Figure 3).

### 3.4 Visual and optical coherence tomography changes related to the type of macular neovascular membrane

**Figure 4:**
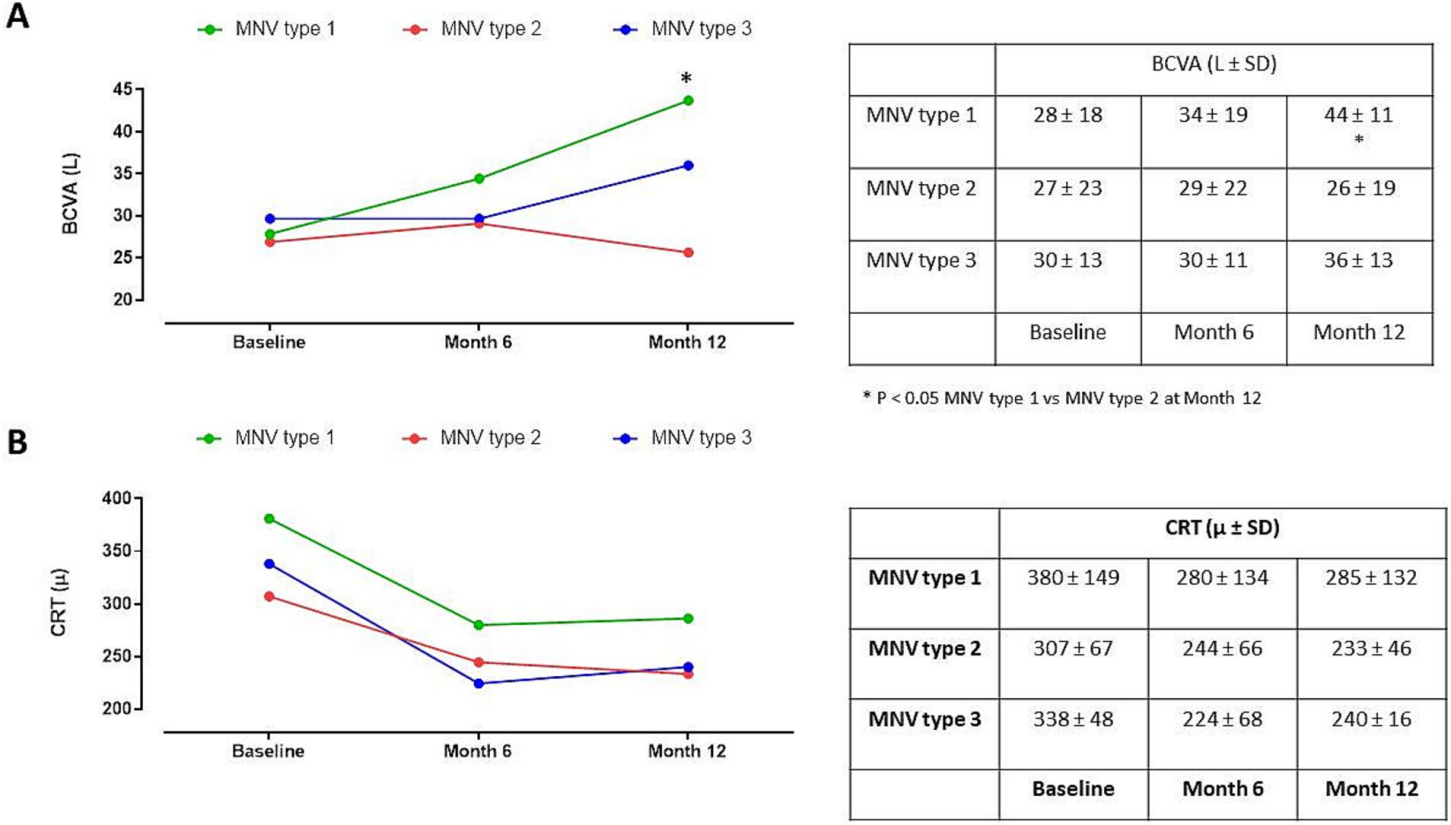
**(A)** Best corrected visual activity (BCVA) changes in patients with macular neovascular membranes (MNV) type 1, 2 and 3 during follow-up. *p < 0.05 MNV type 1 vs. MNV type 2 at Month 12 (RM-ANOVA, followed by Bonferroni’s test). **(B)** Central retinal thickness (CRT) changes in patients classified in MNV type 1, 2 and 3 during follow-up (RM-ANOVA, followed by Bonferroni’s test).

Furthermore, the analysis of BVCA in relation to MNV type (type 1, 2 and 3) did not show substantial changes between groups at baseline and after 6 months (*p > 0.05*), while a significant BVCA improvement was evident in MNV type 1 compared to MNV type 2 (*p < 0.05*) after 12 months (Figure 4A). Moreover, the three MNV groups did not show any CRT modifications at all time points considered (*p > 0.05*) (Figure 4B).

### 3.5 Changes of subretinal hyperreflective material during follow-up

**Figure 5:**
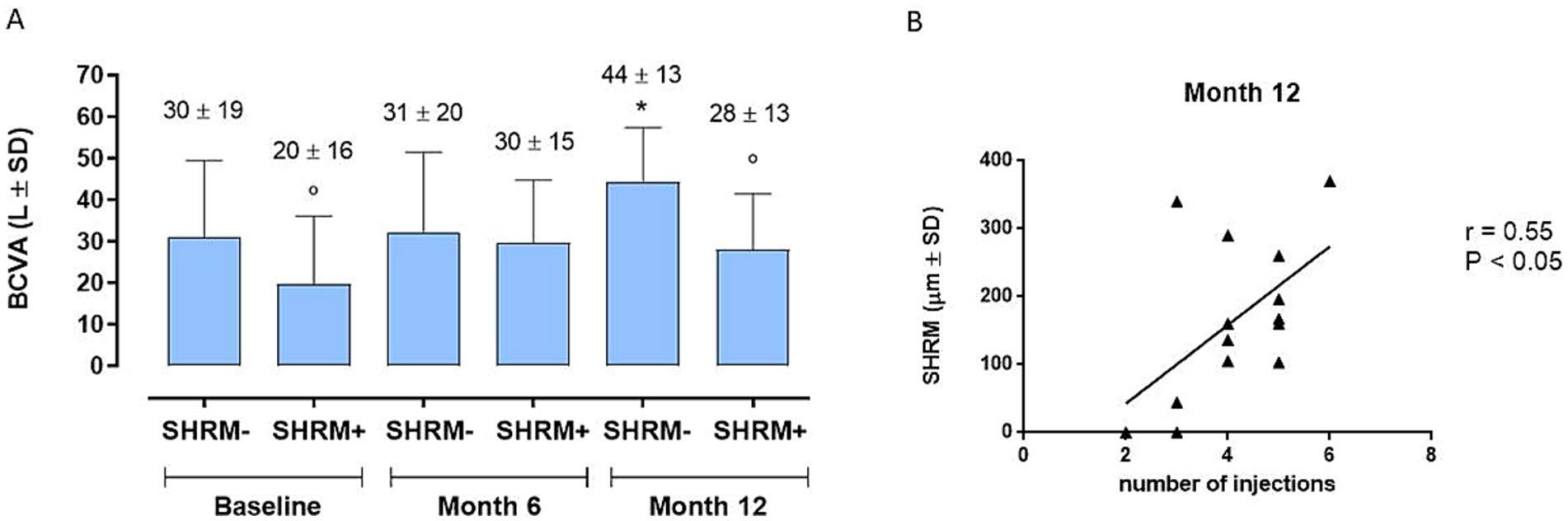
**(A)** Best corrected visual acuity (BCVA) changes in patients with or without baseline subretinal hyperreflective material (SHRM) (SHRM- and SHRM+, respectively) during follow-up. *p < 0.05 vs. Baseline, same group; ° *p* < 0.05 vs. SHRM-, same time point (RM-ANOVA, followed by Bonferroni’s test, Greenhouse–Geisser correction). **(B)** Significant positive correlation (*p* < 0.05) between subretinal hyperreflective material (SHRM) and number of injections at Month 12, confirmed by a significant linear regression analysis (*p* = 0.0429). r = Pearson’s coefficient.

**Figure 6:**
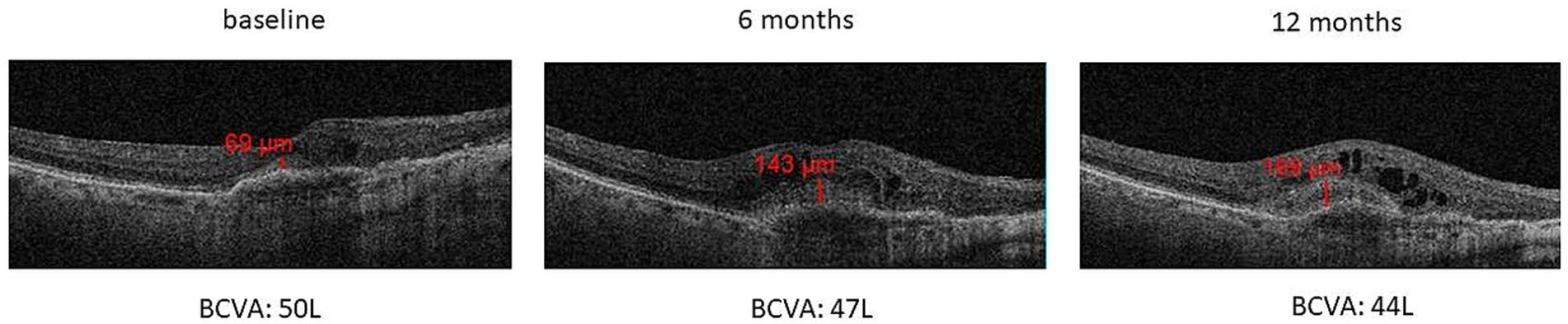
Increase of SHRM thickness with concomitant BCVA decrease during 12-months follow-up in a naïve patient that performed 5 intravitreal injections of Brolucizumab.

Regarding the SHRM analysis, we observed: (a) the presence of SHRM at baseline was correlated with a lower BCVA after 12 months (*p* < 0.05) (Figure 5A); (b) patients with SHRM at baseline and who performed a greater number of IVTs (equal to or greater than 4 IVTs), showed the persistence of thicker SHRM at the end of follow-up (Figure 5B). In the exemplificative Figure 6, it is possible to observe a case of a naïve patient who presented SHRM at baseline, and despite undergoing 5 injections of Brolucizumab, showed a progressive increase in the SHRM thickness, with concomitant BCVA worsening during the follow-up (Figure 6).

## 4 Discussion

In this investigation, the morphological and functional changes observed in real life in 45 eyes with nAMD treated with Brolucizumab, after 1 year of follow-up were analyzed.

This study included two cohorts: treatment naïve (Group 1) subjected to a monthly loading phase of 3 injections of Brolucizumab + Q8/Q12; non-naïve (Group 2), who received a single intravitreal injection followed by a PRN treatment regimen. Both groups analyzed demonstrated significant improvements in retinal function (BCVA) and morphology (CRT) at 12 months, with an average improvement of approximately 10 L ETDRS at the end of the follow-up, and with a significant reduction in fluid accumulation in the various compartments (IR, SR, SRPE) at both 6 and 12 months.

The efficacy of Brolucizumab in BCVA enhancement has been previously explored, with reported outcomes displaying variability across studies (33, 35). Notably, the findings of our investigation align more closely with a real-world study conducted by Bilgic et al., which reported BCVA gains in treatment-naïve patients comparable to ours (36). A visual improvement similar to what was observed in our paper has been described by Scupola A. et al., which however describe a greater visual recovery in naïve patients compared to non-naïve patients (+16.0 ± 4.9 L vs. 10.7 ± 5.9 L), associated with a concomitant greater reduction in macular thickness at the end of follow-up (37). Instead, in the BREW study (35), another real-life study, the safety and efficacy of Brolucizumab in determining a stabilization of vision, and not an improvement, was demonstrated only in a cohort of non-naive patients, and after a rather short follow-up of approximately 4 months.

Our findings underscore the effectiveness of Brolucizumab in managing wet-AMD and suggest its potential for long-term efficacy in stabilizing retinal exudation and fluid accumulation, resulting in improved visual prognosis. As proof of the long-lasting action of the drug, in our cohort we achieved good visual recovery after 1 year of follow-up, performing an average small number of 3.9 + 1.9 IVT (on average 2 IVT after the loading phase), and applying a relaxed loading dose in naïve patients (every 45 days), or a single injection + PRN in non-naïve patients. These data suggest a long-lasting effect of the drug, with good anatomical-functional results obtainable even with a limited number of injections not too close together, thus reducing the risk of intraocular inflammatory phenomen in this regard, in light of the exponential increase in the prevalence of AMD in the coming years as described in multiple studies (1–3), with consequent increase in the global burden of managing AMD itself (1), and in particular of the advanced form (neovascular), our study, although conducted on a small sample, underlines the importance of using long-acting drugs in real life, such as Brolucizumab, in order to reduce the burden through a smaller number of both injections and check-ups.

Another interesting observation arises from the PROBE study, wherein authors demonstrated the potential efficacy of an initial PRN regimen for Brolucizumab therapy, without loading dose. In fact, the authors observed a significant outcome in terms of both BCVA and CRT at the end of the 11-month follow-up, with a mean of 2.2 ± 0.9 injections (38).

It’s conceivable that the smaller size of the Brolucizumab molecule could lead to higher intraocular concentrations, resulting in greater efficacy and duration of pharmacological action (38). However, this characteristic of the drug can determine an increased formation of immune complexes which are the basis of the drug-related immuno-inflammatory reactions, described in multiple studies.

Interesting to note, that however in our study, only one treatment-naïve male patient developed intermediate uveitis. This patient promptly underwent both systemic and topical steroid therapy, with the addition of local mydriatics, showing a rapid and complete visual recovery, demonstrating the importance of early recognition of the initial signs of intraocular inflammation and the consequent timely initiation of the right therapy. An interesting fact that we observed in the management of this patient is the prompt response to steroid therapy with rapid improvement of both the objective and tomographic (OCT) picture, with progressive reduction of the intraretinal edema associated with the neovascular membrane, probably attributable to the decrease in inflammatory component of the disease following the established therapy.

Moreover, at the 12-month follow-up, we observed a significant BCVA improvement in patients with MNV type 1 compared to patients with MNV type 2, without significant differences in terms of CRT. These results are in line with the previous literature data, supporting the evidence that type 1 MNVs are associated with better long term outcomes respect to type 2 MNVs that typically respond quickly to anti-VEGF therapy but are more prone to develop a fibrotic scar (39).

Furthermore, in our study, we observed a significant reabsorption of fluid accumulation in the different retinal compartments (IR, SR, SRPE) during the 12-month follow-up, as already reported in the Hawk and Harrier pivotal studies, and in other real-world studies (16, 23, 40, 41).

Finally, we analyzed the role of a new OCT biomarker, namely SHRM, corresponding to the presence of hyperreflective material in the subretinal space, in relation to the variations in BCVA detected during follow-up. Similarly, to a post-hoc analysis of the Hawk and Harrier trials (25), and what was stated in a recent long-term study by Fang et al. (42), our study confirmed the negative prognostic role of SHRM, being correlated to a lesser or absent visual recovery in patients (both naïve and non-naïve), who showed greater SHRM thickness at baseline. These results are in agreement with the hypothesis that SHRM, corresponding to the accumulation of blood, fibrin and fibro-scar tissue, creates a mechanical barrier between the retina and RPE, thus interfering with metabolic exchanges and normal functionality of the photoreceptors (43).

At the end of our 12-month analysis, we observed that the patients with SHRM at baseline and who performed a greater number of IVTs (equal to or greater than 4 IVTs), showed the persistence of thicker SHRM at the end of follow-up. The progressive increase in the thickness of the SHRM during follow-up could represent a progressive fibro-cicatricial reaction related to the evolution of the disease itself, and which could also be partially accentuated by antiangiogenic therapy with Brolucizumab. In fact, Brolucizumab is a molecule that shows very innovative and particular structural characteristics such as: small dimensions with a molecular mass of only 26 kDa (smaller than other anti-VEGFs), which allow the achievement of a high concentration, bioavailability and persistence at retinal level, resulting in a high VEGF binding capacity which is 11–22 times greater than aflibercept or ranibizumab (44). All this translates into a very rapid and powerful anti-exudative and anti-hemorrhagic action of Brolucizumab, which could on the other hand also determine a more rapid and marked fibrotic reaction. This result suggests that, especially in the more advanced stages of wet AMD, a very careful selection of patients who can actually benefit from further injections is necessary, especially in real-life where we often encounters problems of an economic and organizational nature.

Since a high number of IVT could also lead to the development of extensive subretinal fibrosis. In this regard, it has been widely demonstrated that even during treatment with anti-VEGF drugs, the development of progressive subretinal fibrosis in the sub-foveal area can occur, representing an important unmet clinical need. For example, the CATT study describes the development of fibrotic scars in 24.7% of all treated eyes, regardless of treatment regimen. Particularly, in the CATT, an average growth in size of the neovascular lesion measured at fluorescein angiography (FA) of approximately: +1.6, +1.9 and + 3.0 mm^2^ was observed for monthly bevacizumab, ranibizumab PRN and bevacizumab PRN respectively, therefore showing a continuous growth of fibrovascular tissue. Daniel et al. have observed that the greater risk of developing fibrosis is associated with some basic characteristics of neovascular membrane (NVM), such as: type 2 (predominantly classic) NVM; a larger lesion; increase in foveal retinal thickness; the presence of SF and/or SHRM on OCT (45).

Although this real-life study provides interesting data derived from the daily management of nAMD, it is characterized by some limitations to underline such as: the retrospective design, the relatively small size of the cohort and the short-term follow-up.

## 5 Conclusion

In conclusion, despite the limitations of the study, our analysis highlights the efficacy and prolonged effect of Brolucizumab therapy in the treatment of nAMD, both in naïve and non-naïve patients, with a good safety profile. Furthermore, this paper underlines the importance of careful selection of the patient to be treated, also considering the negative prognostic role of the possible presence of SHRM at baseline. However, prospective studies with longer follow-up and larger cohorts are needed to better study the long-term effects and any adverse reactions attributable to Brolucizumab therapy.

## Data Availability

The original contributions presented in the study are included in the article/supplementary material, further inquiries can be directed to the corresponding author.
